# Genetic ablation of the mammalian sterile-20 like kinase 1 (Mst1) improves cell reprogramming efficiency and increases induced pluripotent stem cell proliferation and survival

**DOI:** 10.1016/j.scr.2017.02.011

**Published:** 2017-04

**Authors:** Abigail Robertson, Tamer M.A. Mohamed, Zeinab El Maadawi, Nicholas Stafford, Thuy Bui, Dae-Sik Lim, Elizabeth J. Cartwright, Delvac Oceandy

**Affiliations:** a Division of Cardiovascular Sciences, The University of Manchester, Manchester Academic Health Science Centre, Manchester, United Kingdom; b J. David Gladstone Research Institutes, San Francisco, CA, USA; c Faculty of Pharmacy, Zagazig University, Egypt; d Department of Histology and Cell Biology, Faculty of Medicine, Cairo University, Egypt; e Department of Biological Sciences, KAIST, Daejon, Republic of Korea

**Keywords:** Hippo pathway, Reprogramming, iPSC, Mst1, Cell proliferation, iPSC, induced pluripotent stem cells, Mst1, mammalian sterile-20 like kinase 1, Oct4, octamer-binding transcription factor 4, Sox2, sex determining region Y box 2, Klf 4, kruppel-like factor 4, Tbx3, T-box 3, Utf1, undifferentiated embryonic cell transcription factor 1, Lats, large tumour suppressor homologue, Sav1, Salvador homologue 1, Mob1, Mps one binder kinase activator-like 1, YAP, Yes-associated protein, WT, wild type, HIF1a, hypoxia inducible factor 1-alpha, Taz, WW domain containing transcription regulator 1, MEF, mouse embryonic fibroblast, Bax, Bcl2 associated X, Bim, Bcl2-like protein 11, Bcl2, B-cell lymphoma 2

## Abstract

Adult fibroblasts can be reprogrammed into induced pluripotent stem cells (iPSC) for use in various applications. However, there are challenges in iPSC generation including low reprogramming efficiency, yield, cell survival and viability. Since the Hippo signalling pathway is a key pathway involved in regulating cell proliferation and survival, we here test whether modification of the Hippo pathway will enhance the efficiency of iPSC generation and improve their survival.

The Hippo pathway was modified by genetic ablation of the mammalian sterile-20 like kinase 1 (Mst1), a major component of the pathway. Using adult skin fibroblasts isolated from Mst1 knockout mice (Mst1^−/−^) as a source of iPSC we found that genetic ablation of Mst1 leads to significantly increased reprogramming efficiency by 43.8%. Moreover, Mst1^−/−^ iPSC displayed increase proliferation by 12% as well as an increase in cell viability by 20% when treated with a chemical hypoxic inducer. Mechanistically, we found higher activity of YAP, the main downstream effector of the Hippo pathway, in iPSC lacking Mst1.

In conclusion, our data suggests that Mst1 can be targeted to improve the efficiency of adult somatic cell reprogramming as well as to enhance iPSC proliferation and survival.

## Highlights

• Genetic deletion of Mst1 increases the efficiency of cell reprogramming.
• iPSC lacking Mst1 displays higher proliferation rate than WT iPSC.
• In response to chemical hypoxia Mst1^−/−^ iPSC demonstrates higher survival.

## 1 Introduction

Induced pluripotent stem cells (iPSC) have been used in an increasing number of applications since Takahashi and Yamanaka (Takahashi and Yamanaka, 2006) first demonstrated the reprogramming of adult somatic cells to produce iPSC. In combination with gene editing technology iPSC have been widely used to model human genetic diseases (Avior et al., 2016), providing a useful alternative to animal models. iPSC-derived cells are also valuable tools in drug discovery since these cells, which can carry phenotypes of a particular disease, can be used in high-throughput screening to find pharmacological compounds that may correct the phenotypes (Avior et al., 2016). Finally, iPSC have emerged as an attractive source of cells for regenerative medicine: the first clinical trial to treat macular degeneration using iPSC-derived retinal epithelium is ongoing (Kimbrel and Lanza, 2015, Reardon and Cyranoski, 2014).

Improving cell reprogramming efficiency is therefore a very important focus of research. A number of studies have been centred on the identification of new reprogramming enhancers that can be used to improve reprogramming efficiency if added to the Yamanaka factors: Oct3/4, Sox2, Klf4, and Myc (OSKM). Several studies have identified factors that can improve cell reprogramming. These include several different types of molecules such as transcription factors, for example TBX3 (Han et al., 2010) and UTF1 (Zhao et al., 2008), oncogenes (e.g. Ras (Kwon et al., 2015)) and micro RNAs (for example miR-294, 295 and 291-3p (Judson et al., 2009)). However, most of these factors are not easily targetable using small molecule/pharmacological compounds, thus identification of novel reprogramming enhancers that are pharmacologically targetable would be very useful.

Properties of the starting adult somatic cells are critical for the success of cell reprogramming. Several aspects are known to contribute to the reprogramming efficiency including cell type, age of cell donor and the differentiation stage of the starting cells. In addition, the proliferation rate is also essential. Actively proliferating cells are more easily reprogrammed than cells in senescence (Utikal et al., 2009).

In this study we target a component of the Hippo pathway, the mammalian ste-20 like kinase 1 (Mst1), to improve cell reprogramming efficiency. The highly conserved Hippo pathway is an intrinsic regulator of organ size during development and is a major regulator of cell proliferation and survival (Yu and Guan, 2013). The core components of this pathway consist of kinases (Mst1/2 and Large tumour suppressor homologue (Lats1/2)) as well as adaptor molecules Salvador homologue (Sav1) and Mps one binder kinase activator-like 1 (MOB1) (Yu and Guan, 2013). In its active state the Hippo pathway will trigger phosphorylation of its effector Yes-associated protein (YAP) leading to cytoplasmic retention and inactivation. Thus, inhibition of core components of the Hippo pathway increases YAP activity, and hence enhances cell proliferation and reduces cell death/apoptosis (Yu and Guan, 2013).

Here we use adult somatic cells (skin fibroblasts) isolated from mice with genetic ablation of the Mst1 gene (Mst1^−/−^ mouse) to examine if inhibition of Mst1 will improve reprogramming efficiency and increase survival of the resulting iPSC.

## 2 Materials and methods

### 2.1 Isolation and culture of adult skin fibroblasts

Mice with genetic deletion of the Mst1 gene (Mst1^−/−^) were used in this study. Generation of Mst1^−/−^ mice has been described elsewhere (Oh et al., 2009). Animal studies were performed in accordance with the United Kingdom Animals (Scientific Procedures) Act 1986 and were approved by the University of Manchester Ethics Committee. Skin biopsies were taken from ear snips and washed in 70% ethanol and then in PBS. The fur was removed and the biopsies were cut into small pieces. The skin fragments were then placed underneath sterile glass coverslips in a 6-well plate to reduce movement and were cultured in Dulbecco's Modified Eagle Medium (DMEM) supplemented with 20% FBS, non-essential amino acids (Gibco), 100 IU/ml penicillin, 100 μg/ml streptomycin and 2.5 μg/ml amphotericin *B. media* was changed every two days until skin fibroblasts could be seen appearing from the biopsies. Once cells reached confluency skin fibroblasts were split and transferred to larger cell culture flasks.

### 2.2 Generation of iPSC

10 μg of the STEMCCA4-lox-P vector (Sommer et al., 2009) (a kind gift from Dr. Gustavo Mostoslavsky, Boston) and 1 μg each of packaging and envelope plasmids were transfected into HEK293 cells using lipofectamine 2000 reagent (ThermoFisher). 24 h after transfection, the media was discarded and replaced with fresh media. On the second and third day the conditioned media containing lentivirus particles was collected for transducing skin fibroblasts. A small aliquot (100 μl) of conditioned medium was collected for lentiviral titre quantification using the LV Lentiviral Titre kit (Mo Bi Tec).

Wild type and Mst1^−/−^ skin fibroblasts were plated at a density of 20,000 cells per well of a 12-well plate. The cells were then incubated with the lentivirus containing media supplemented with Polybrene (Millipore) for 24 h. After 24 h the lentivirus containing media was removed and cells were then maintained in DMEM with 10% FBS for 7 days. Then cells were transferred to 0.1% gelatine coated plates containing Mitomycin C-deactivated mouse embryonic fibroblasts (MEF). From this point the cells were maintained in DMEM supplemented with 20% FBS and 1 ng/ml of leukaemia inhibitory factor (LIF) (Invitrogen). For iPSC colony counting, colonies were stained for alkaline phosphatase activity using the Leukocyte Alkaline Phosphatase kit (Sigma).

### 2.3 RNA isolation and qPCR analysis

RNA was extracted from monolayer cells using PureLink RNA mini kit (ThermoFisher) following a protocol recommended by the manufacturer. RNA samples were then treated with DNase (Sigma) to remove contaminating DNA. For quantitative real time PCR, DNase treated RNA samples were converted to cDNA using a High-Capacity cDNA reverse transcription kit (Applied Biosystems). Subsequent qPCR analysis was then performed using Brilliant III SYBR green qPCR kit (Agilent Technologies). We used the QuantiTect Primer Assays (Qiagen) to detect expression of pluripotency markers (Nanog, Sox2, Oct4).

### 2.4 Western blots

Cells were washed in PBS and the total protein extracts were collected in RIPA buffer (1 × PBS, 1% IGEPAL CA-630, 0.5% sodium deoxycholate, 0.1% SDS, 0.5 mM PMSF, 500 ng/ml Leupeptin, 1 mg/ml Aprotinin, 2.5 mg/ml Pepstatin A). The bicinchoninic acid (BCA) assay kit (Pierce) was used to determine protein concentration. Western blot analyses were performed using a method described previously (Omede et al., 2016). Primary antibodies used were anti-Mst1, anti-Mst2, anti-Lats1, anti-phospho-Lats1, anti-Mob1, anti-Sav1, anti-Nanog, anti-Sox2, anti-Klf4 (all from Cell Signaling), anti-GFP, anti-GAPDH and anti-β-actin (from Abcam). HRP-conjugated antibodies (Cell Signaling) were used as secondary antibodies.

### 2.5 EdU incorporation assay

We used the Click-It EdU imaging kit (ThermoFisher) to measure cell proliferation rate. Cells were plated at a density of 5000 cells per well in a 24-well plate containing sterile cover slips and were labelled with EdU labelling reagent. After 24 h cells were washed with PBS and fixed with 4% paraformaldehyde. EdU incorporation was detected using the antibody (supplied within the kit) following the manufacturer's recommended protocol. The percentage of EdU positive cells was calculated by counting the number of cells with positive EdU staining divided by the total number of cells.

### 2.6 Analysis of cell survival and apoptosis

Cells were treated with 250 μM CoCl_2_ for 16 h to mimic cellular hypoxic condition as described elsewhere (Wu and Yotnda, 2011). Cell viability was measured using 0.4% Trypan Blue solution (Sigma) and viable cells were counted using the Countess Automated Cell Counter (Life Technologies). For caspase assay, cells were lysed using a cell lysis buffer (Promega) and then treated with Caspase-Glo 3/7 Reagent (Promega) for 2 h in the dark as per the manufacturer's guidelines. The luminescence signal was measured using a FLUOstar Omega plate reader (BMG Labtech).

### 2.7 Analysis of YAP activity

We used a luciferase based assay developed previously (Tian et al., 2010) to monitor YAP activity. We used two plasmids, one containing GAL4-TEAD construct, a gift from Dr. Kunliang Guan (Addgene plasmid #24640) and the other containing UAS-luciferase cassette, a gift from Dr. Liqun Luo (Addgene plasmid #24343) (Potter et al., 2010). To generate adenoviruses we cloned the GAL4-TEAD or the UAS-luciferase fragments into the pENTR11 shuttle vector and then transferred these to the adenovirus vectors pAd-DEST or pAd-CMV-DEST (Invitrogen) using the Gateway vector system (Invitrogen) producing the pAd-CMV-GAL4-TEAD and pAd-UAS-luciferase. Adenovirus was generated by transfecting the adenovirus plasmids into HEK293 cells.

To detect the YAP subcellular location, we used the GFP-YAP construct, a gift from Dr. Marius Sudol (Addgene plasmid #17843) (Basu et al., 2003). Adenovirus expressing the GFP-YAP construct was generated using the Gateway system as described above.

### 2.8 Data analysis

Data were expressed as mean ± SEM. Student's *t*-test or one way ANOVA were used to test statistical significance. The probability level for statistical significance was set at *p* < 0.05.

## 3 Results

### 3.1 Mst1^−/−^ adult skin fibroblasts display a higher proliferation rate

**Fig. 1:**
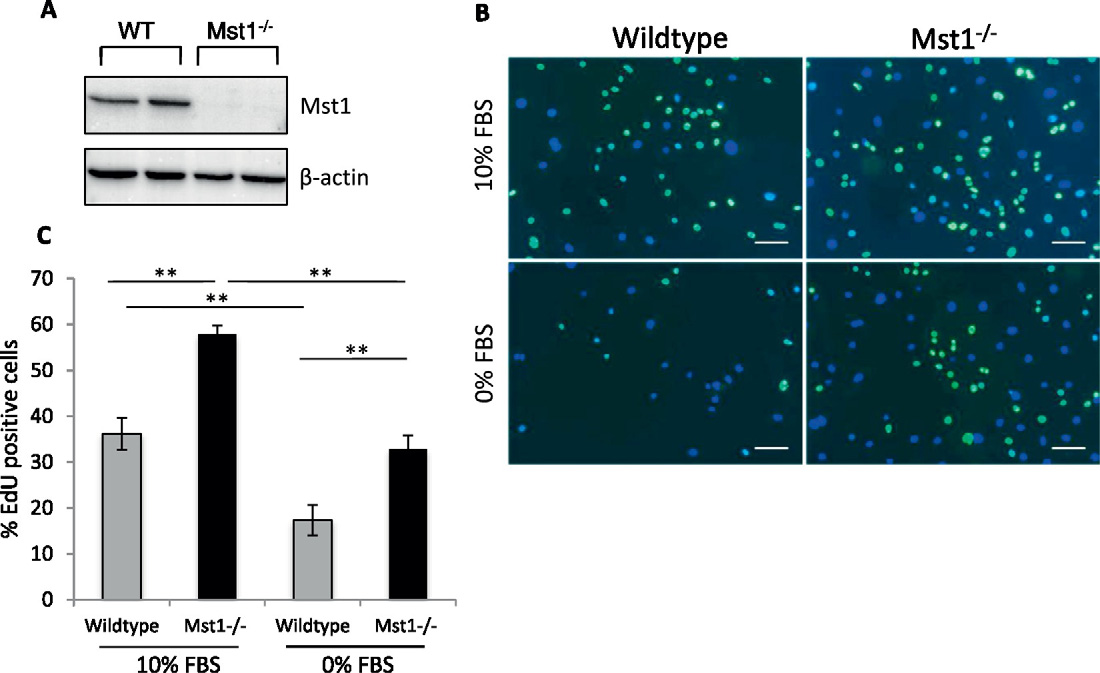
Adult skin fibroblasts isolated from Mst1^−/−^ mice exhibit a higher proliferation rate. A) Western blot analysis showing the complete absence of Mst1 in Mst1^−/−^ skin fibroblasts. B) Representative images of EdU incorporation assay. Nuclei were stained for EdU incorporation (green) and DAPI (blue). Scale bars = 100 μm. C) Quantification of EdU positive cells showed that adult skin fibroblasts lacking Mst1 displayed a higher proliferation rate than wild type cells (**P < 0.01, *n* = 3 biological replicates, i.e. fibroblasts were isolated from 3 independent mice in each group, one way ANOVA followed by post-hoc multiple comparisons).

Adult skin fibroblasts were cultured from the ear skin tissue biopsies of 12 week old Mst1^−/−^ and wild type (WT) mice. We confirmed the absence of Mst1 expression in the knockout fibroblasts by Western blot analysis (Fig. 1A). We then used an EdU incorporation assay to examine the effects of Mst1 ablation on cell proliferation rate. EdU is incorporated into the nucleus of cells undergoing proliferation and was detected using a fluorescence labelled antibody. We examined the level of fibroblast proliferation in normal medium containing 10% FBS and in serum-starved medium (0% FBS). We found that Mst1^−/−^ fibroblasts exhibited a significantly higher proliferation rate compared to WT fibroblasts in both normal and serum-starved medium (Fig. 1B–C).

### 3.2 Mst1 ablation increases reprogramming efficiency

**Fig. 2:**
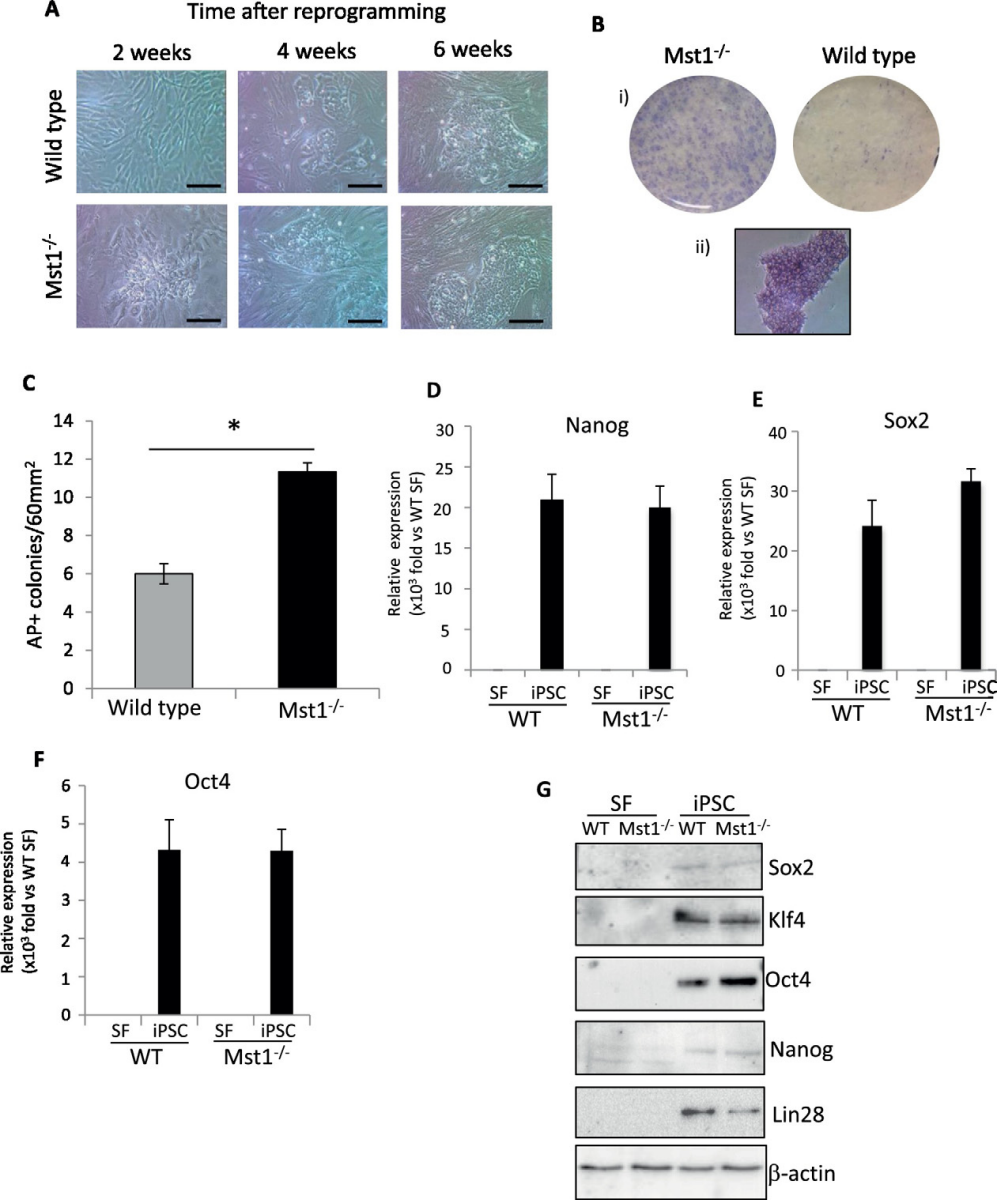
Ablation of Mst1 enhances reprogramming efficiency. A) Representative images of mouse skin fibroblasts following reprogramming using STEMCCA lentivirus. iPSC colonies appeared at an earlier time point in Mst1^−/−^ cells. Scale bars = 100 μm. B) i) Images of iPSC colonies following staining for alkaline phosphatase 4 weeks after reprogramming. ii) Example of iPSC colony in higher magnification following alkaline phosphatase staining. C) Quantification of alkaline phosphatase positive colonies revealed a significant higher number of iPSC colonies in the Mst1^−/−^ group compared with wild type (**P < 0.01, *n* = 3 biological replicates, i.e. fibroblasts were isolated from 3 independent mice in each group). Expression of pluripotency markers: Nanog (D), Sox2 (E) and Oct4 (F) were examined by quantitative real time RT-PCR. Expression of pluripotency markers were significantly higher in iPS cells compared to their original skin fibroblasts population (***P < 0.01, *n* = 3 independent cell cohorts in each group), but there was no difference between Mst1^−/−^ iPSC vs WT iPSC. G) Western blot analysis confirmed the expression of pluripotency markers: Sox2, Klf4, Oct4, Nanog and Lin28 in both Mst1^−/−^ and WT iPSC. All of the statistical analyses in this figure were performed using Student's *t*-test.

To investigate if deletion of Mst1 modifies the efficiency of cell reprogramming we transduced skin fibroblasts with lentivirus expressing the Yamanaka factors (Oct3/4, Sox2, Klf4, and cMyc). We used a single lentiviral cassette expressing all four of the factors (STEMCCA cassette) (Sommer et al., 2009). Representative images showing the morphology of the cells during the course of reprogramming are shown in Fig. 2A. After 4 weeks of reprogramming, the number of iPSC colonies was analysed. iPSC colonies were stained for alkaline phosphatase activity, which is a marker of pluripotency. Stained colonies in multiple 60 mm^2^ areas were counted and revealed a significantly higher number of colonies present in the Mst1^−/−^ (48% higher) compared to the wild type mouse IPSC (Fig. 2B–C).

To confirm the pluripotency of the iPSC colonies the expression of pluripotency genes Nanog, Sox2, and Oct4 were assessed using qRT-PCR (Fig. 2D–F). Both Mst1^−/−^ and WT mouse iPSC showed significantly higher expression of these pluripotency genes compared to their skin fibroblast populations. However, there was no significant difference between Mst1^−/−^ and WT iPSC (Fig. 2D–F). We also examined protein expression levels of the pluripotency markers using Western blot and showed expression of Nanog, Sox2, Klf4, Oct4 and Lin28 in both Mst1^−/−^ and WT iPSC, which were absent in fibroblasts (Fig. 2G). Together our data indicated that by using the four reprogramming factors, fibroblasts lacking Mst1 showed a higher efficiency of reprogramming.

### 3.3 Proliferation rate is higher in Mst1^−/−^ iPSC

**Fig. 3:**
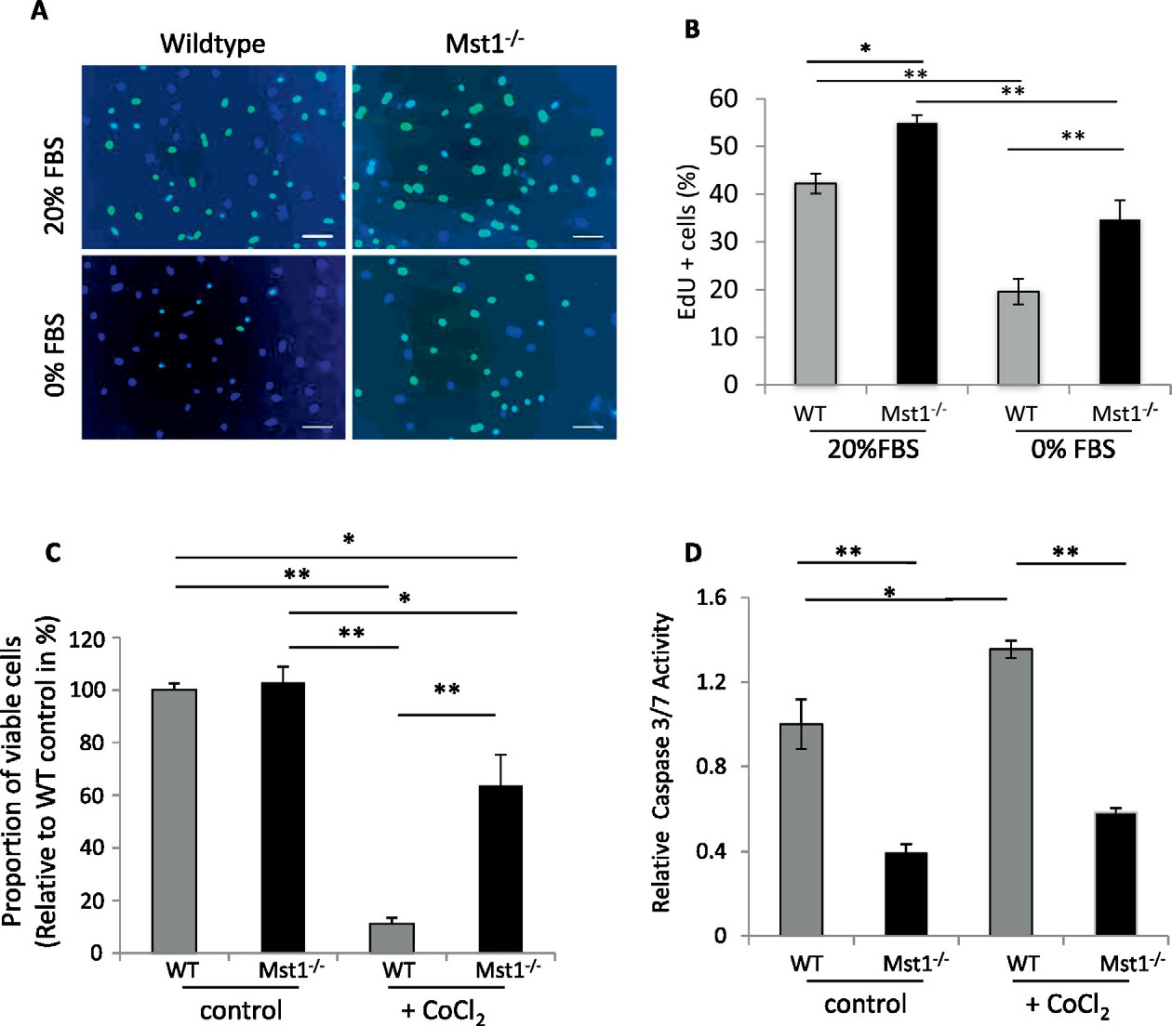
Mst1^−/−^ iPSC exhibit higher proliferation rates and survival. A) Images of EdU incorporation assay on Mst1^−/−^ and WT iPSC in normal media containing 20% FBS and in starving media (0% FBS). B) Quantification of EdU positive cells indicated that iPS cells lacking Mst1displayed higher proliferation rate in both normal and starving media (*P < 0.05, **P < 0.01, *n* = 3 independent iPSC in each group, one way ANOVA followed by post-hoc multiple comparisons). C) Measurement of cell viability using Trypan blue staining demonstrated that following treatment with CoCl_2_ for 16 h Mst1^−/−^ iPSC showed higher viability compared to WT iPSC (**P < 0.01, *n* = 3 independent iPSC populations, one way ANOVA followed by post-hoc multiple comparisons). D) Analysis of Caspase 3/7 activity indicated that Mst1^−/−^ cells displayed lower caspase activity in both basal condition and after treatment with CoCl_2_ for 16 h (*P < 0.05, **P < 0.01, *n* = 3 independent iPSC populations, one way ANOVA followed by post-hoc multiple comparisons).

Since Mst1 ablation increased proliferation in the skin fibroblasts prior to reprogramming, we then analysed the proliferation rate of Mst1^−/−^ iPS cells. Using an EdU incorporation assay we found a significantly higher number of EdU positive cells in the Mst1^−/−^ iPSC compared to WT iPSC (Fig. 3A–B). When we cultured iPSC in starving medium (0% FBS) we observed similar phenotype with a higher proliferation rate in Mst1^−/−^ iPSC. The data suggested that ablation of Mst1 not only enhances reprogramming efficiency but also increases iPSC proliferation.

### 3.4 iPS cells lacking Mst1 shows enhanced viability and survival

Components of the Hippo pathway are known to regulate cell apoptosis and survival. In particular, Mst1 has been shown to have a pro-apoptotic effect in a number of cell types (Ardestani et al., 2014, Del Re et al., 2014, Ura et al., 2001). We therefore analysed the viability and survival of Mst1^−/−^ iPSC when exposed to a toxic stimulus. We treated cells with cobalt chloride (CoCl_2_) 250 μM for 16 h. CoCl_2_ treatment mimics cellular hypoxic conditions by inducing hypoxia inducible factor 1-alpha (HIF1α) (Wu and Yotnda, 2011). We measured the percentage of viable cells and the caspase 3/7 activity following CoCl_2_ treatment. Under basal conditions there was no difference between the viability of Mst1^−/−^ and wild type mouse iPSC. However, Mst1^−/−^ mouse iPSC had a significantly higher proportion of viable cells after hypoxic treatment (64% of non-treated WT cells) than the wild type mouse iPSC (12% of non-treated WT cells) (Fig. 3C). We also measured caspase 3/7 activity as a marker of cell apoptosis. In Mst1^−/−^ mouse iPSC the levels of caspase 3/7 were significantly lower than in the wild type cells both at basal condition and after CoCl_2_ treatment (Fig. 3D). The data suggested that ablation of Mst1 increased cell viability and reduced apoptosis in iPSC following chemical hypoxia.

### 3.5 YAP activity is increased in iPSC lacking Mst1

**Fig. 4:**
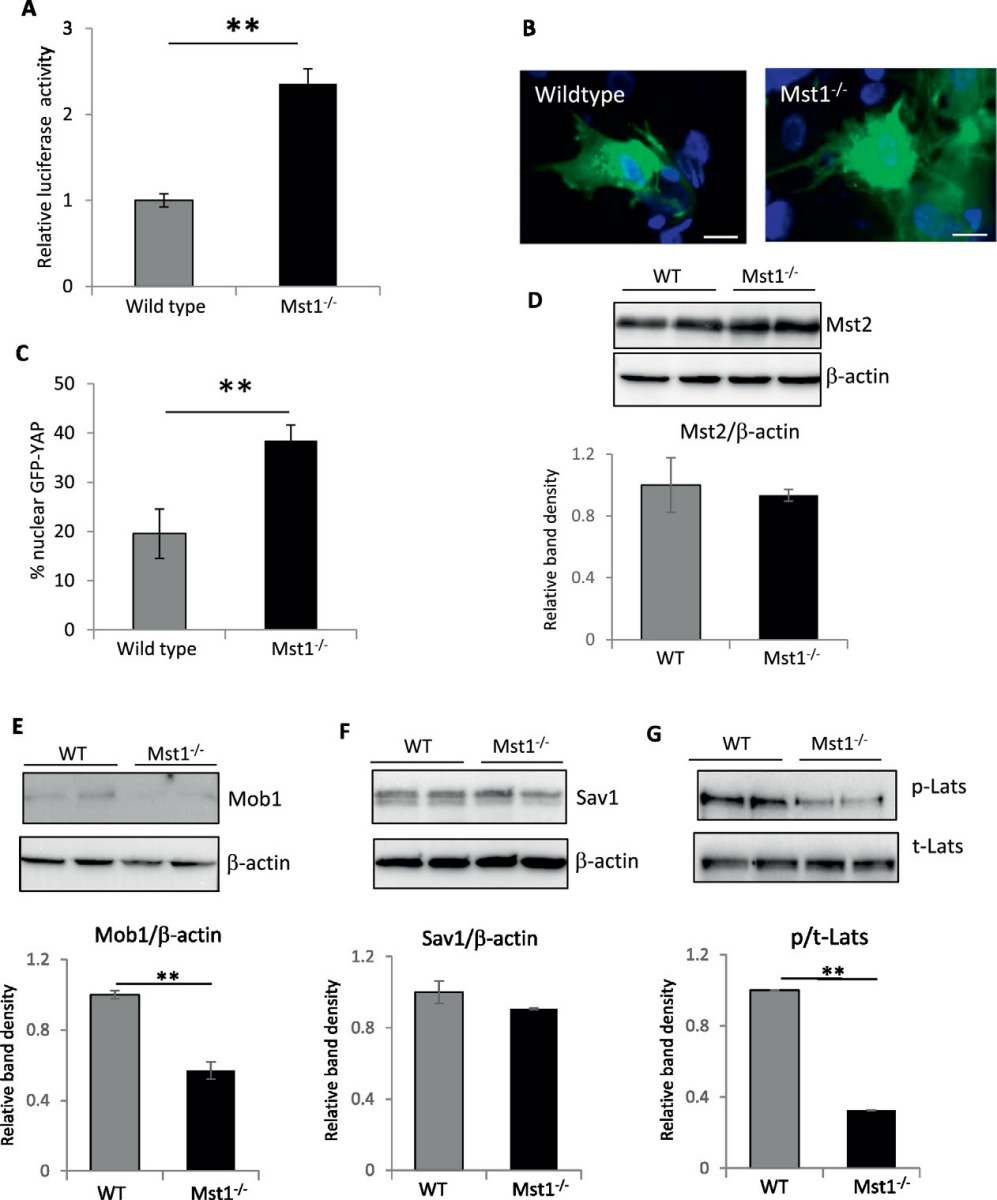
YAP activity is increased in Mst1^−/−^ iPSC. A) Analysis of YAP activity using a YAP luciferase reporter showed significantly (**P < 0.01) higher YAP activity in Mst1^−/−^ iPS cells vs WT. B) Examples of fluorescent microscopy images of iPSC transduced with adenovirus expressing GFP-YAP. Scale bars = 10 μm. C) Quantification of cells with YAP nuclear localization showed a significantly higher number of Mst1^−/−^ iPSC with nuclear YAP compared to WT (**P < 0.01, *n* = 3 independent iPSC populations in each group). Western blot and analysis of band density showing expression of D) Mst2, E) Mob1, F) Sav1 and G) phosphorylated/total Lats1. Mst1^−/−^ iPS cells displayed significantly reduced levels of Mob1 and phospho/total Lats1 (**P < 0.01, *n* = 3 independent iPSC populations). All of the statistical analyses in this figure were performed using Student's *t*-test.

The major downstream effector of the Hippo signalling cascade is the transcription co-activator YAP. Mst1 induces activation of Lats1 by phosphorylation, which subsequently leads to YAP phosphorylation and thereby cytoplasmic retention and inactivation (Yu and Guan, 2013). To investigate the activity of this pathway we analysed YAP activation and subcellular localization in the iPSC using a YAP-luciferase reporter system and a GFP-YAP construct. We generated adenoviruses to enable efficient gene transfer of these reporter systems into the iPSC. Using these viruses we detected significantly higher YAP activity in the Mst1^−/−^ iPSC compared to WT iPSC (Fig. 4A). In keeping with this data, when we detected YAP sub-cellular localization using the GFP-YAP construct we observed a higher proportion of cells with nuclear YAP in the Mst1^−/−^ iPSC than in WT (Fig. 4B–C).

Furthermore, we analysed expression of components of the Hippo pathway by Western blot. We found a significant reduction in Lats1 phosphorylation and Mob1 expression, whereas total Mst2 and Salvador (Sav1) expressions remained unchanged (Fig. 4D–G). Overall, our data showed a consistent regulatory mechanism by Mst1, in which ablation of this kinase led to a reduction in Lats1 phosphorylation and an increase in YAP activity.

## 4 Discussion

The key findings of this study are: i) genetic deletion of Mst1 increases the efficiency of skin fibroblasts reprogramming to iPSC and ii) iPSC lacking Mst1 exhibits higher proliferation rates and increased survival when treated with chemical hypoxic agents. Mst1 is a core member of the Hippo signalling pathway, which is known as a major regulator of cell proliferation, apoptosis and survival (Yu and Guan, 2013). Here we demonstrated that ablation of Mst1 in iPSC resulted in the activation and nuclear translocation of YAP, the main Hippo effector, leading to an induction in proliferation and reduction of apoptosis.

Induced pluripotent stem cells have emerged as powerful tool for many applications including disease modelling, drug screening and cell based therapies (Takahashi and Yamanaka, 2016). Therefore, increased efficiency of cell reprogramming is beneficial as it could help reduce the time required to produce iPSC. In addition, an enhanced proliferation rate would also be useful as in many applications the number of cells is critical for the successful use of iPSC.

Several aspects have been regarded as contributing factors towards reprogramming efficiency including the type and the differentiation stage of the starting adult somatic cells as well as their proliferation rate. Some particular cell types exhibit higher reprogramming efficiency, for example mesenchymal stem cells (MSC) are easier to reprogramme than keratinocytes from the same individual, possibly due to the higher methylation state within key pluripotency genes in the keratinocytes (Streckfuss-Bomeke et al., 2013). Likewise, the differentiation stage of the starting cells is also important: Eminli and colleagues have shown that undifferentiated haematopoietic cells are far more efficiently reprogrammed compared to terminally differentiated B and T cells (Eminli et al., 2009). In addition to these factors, recent observations have also shown that proliferation rate and/or cellular senescence of the starting cells as well as the age of donor cells appear to play essential roles in determining reprogramming efficiency (Utikal et al., 2009, Banito et al., 2009, Kawamura et al., 2009, Li et al., 2009, Marion et al., 2009).

We therefore reasoned that modification of the proliferation rate of the starting cells by modulating a key signalling pathway that regulates cell proliferation might be used as a strategy to improve cell reprogramming. We focused on the Hippo signalling since this pathway is one of the primary regulators of cell proliferation, cell survival and organ size control (Yu and Guan, 2013, Johnson and Halder, 2014). Components of the Hippo pathway modulate proliferation in various cell types. For example, Mst1 gene silencing induces proliferation of glioma cells (Chao et al., 2015), whereas in mouse intestinal epithelium ablation of both Mst1 and Mst2 resulted in cell expansion and proliferation (Zhou et al., 2011). The other central components of the Hippo pathway, Lats1/2 are also essential in mediating cell proliferation. Knockout of Lats1 and Lats2 increases proliferation of biliary epithelium and hepatoblasts (Yi et al., 2016), in contrast, overexpression of Lats1 reduces proliferation of human breast cancer cells MCF-7 (Xia et al., 2002). TAZ, one of the downstream effectors of the Hippo pathway, also promotes cell proliferation (Lei et al., 2008).

In this study we focused on targeting the Mst1 kinase. In the Hippo signalling cascade Mst1 acts as a negative regulator of the proliferative signal by phosphorylating Lats1/2 and eventually results in YAP phosphorylation and hence inactivation (Yu and Guan, 2013, Johnson and Halder, 2014). In the present study we found that genetic ablation of Mst1 significantly enhanced the reprogramming efficiency of adult mouse skin fibroblasts to iPSC. The improvement of reprogramming efficiency might be, at least in part, due to the increased proliferation rates of the starting somatic cells as the skin fibroblasts lacking Mst1 displayed higher proliferation rates in both normal and starving medium. This is consistent with a previous report showing that highly proliferative early passage mouse embryonic fibroblasts (MEFs) displayed higher efficiency of reprogramming compared to late passage MEFs, which display a lower proliferation rate (Utikal et al., 2009). In agreement with this, various observations have demonstrated that in less proliferative cells or in senescent cells the reprogramming efficiency is low (Banito et al., 2009, Kawamura et al., 2009, Li et al., 2009, Marion et al., 2009). Activation of the p53 pathway in such cells is thought to be the underlying mechanism for the low reprogramming efficiency (Kawamura et al., 2009).

Interestingly, the Mst1^−/−^ iPSC also demonstrated a higher proliferation rate compared to WT iPSC indicating that the reprogrammed cells retained the properties of the source cells in terms of proliferation rate. Analysis of the Hippo signalling pathway in these cells suggested a marked activation of YAP and reduction of Lats1 phosphorylation and Mob1 expression, all of which fit consistently with the current understanding of Hippo pathway regulation: ablation of Mst1 will result in Lats1 inactivation and hence an increase in YAP activity and nuclear translocation. Importantly, the fact that Mst1^−/−^ iPSC retain the high proliferation phenotype might become an additional benefit to the higher reprogramming efficiency since it will increase the speed of iPSC generation.

The second important phenotype that we studied was cell survival. We analysed this phenotype because the Hippo pathway is also a major regulator of cell apoptosis and survival. Components of this pathway including Mst1, Lats1/2 and YAP are implicated in the regulation of cell apoptosis. Mst1 for example, has been described as a strong apoptosis inducer in many cell types (Ardestani et al., 2014, Del Re et al., 2014, Ura et al., 2001). Lats1/2, the downstream effector of Mst1, also promotes apoptosis (Xia et al., 2002, Aylon et al., 2010) whereas inhibition of this molecule attenuates apoptotic cell death (Matsui et al., 2008). We found here a consistent phenotype, in which the Mst1^−/−^ iPSC displayed lower apoptosis and hence higher survival when challenged with chemical hypoxia by treatment with CoCl_2_. This phenotype could be attributable to a decrease in Mst1, higher YAP activity or both. Mst1 may promote cell apoptosis by activating pro-apoptotic molecules, such as caspases (Ura et al., 2001), Bax (Del Re et al., 2014) or BIM (Ardestani et al., 2014). Whilst, YAP promotes cell survival by inducing expression of anti-apoptotic genes, such as Bcl2 (Song et al., 2016) and survivin (Bai et al., 2012). In addition to the high proliferative capacity, this phenotype might be beneficial to improve the production rate of the iPSC.

Perhaps the most interesting aspect of this study is the possible practical application of the findings. Although a number of studies have identified factors that can promote reprogramming efficiency, few have suggested factors that are likely to be pharmacologically targetable. Whilst targeting a particular molecule may indeed be attained by genetic approaches, e.g. using RNA interference or overexpressing constructs, the most ideal strategy to improve the efficiency of cell reprogramming would be by using pharmacological compounds. Inhibition of Mst1 by a small molecule is achievable since a recent report has described the identification of novel pharmacological inhibitor of Mst1 and Mst2 by using large scale library screening (Fan et al., 2016). This new compound successfully blocked phosphorylation of Mst's targets and induces YAP activation in various cell types. Treatment with this inhibitor also induced proliferation of liver cells in vivo. Given the possibility to inhibit Mst1/2 activity pharmacologically, targeting this molecule may become an attractive approach to improve the reprogramming of adult somatic cells to iPSC and to increase the efficiency of iPSC production. Therefore, in future it will be very interesting to examine the effects of Mst1 inhibitor treatment on iPSC reprogramming efficiency as well as on iPSC proliferation and survival.

## 5 Conclusion

In conclusion, the results of this study establish Mst1 as a possible target to enhance the efficiency of adult somatic cells reprogramming to iPSC as well as to increase the proliferation and survival of the resulting iPSC. This might be beneficial for potential application to improve the efficiency and the rate in generating iPSC.
